# Effects of continuous variation in vertical and lateral herkogamy on reproductive success in *Euphorbia fischeriana* (Euphorbiaceae)

**DOI:** 10.1002/ece3.9836

**Published:** 2023-02-15

**Authors:** Xiang Zhao, Guang Yang, Qinzheng Hou, Wenrui Min, Taihong Wang, Xiaoyan Bao

**Affiliations:** ^1^ College of Life Sciences Northwest Normal University Lanzhou China

**Keywords:** continuous variation, *Euphorbia fischeriana*, herkogamy, reproductive success

## Abstract

Continuous variation in herkogamy has been well reported, however, less attention has been paid to the phenomena that the consecutive expression of two types of herkogamy in the same flower. *Euphorbia fischeriana,* which have both vertical and lateral herkogamy, show vertical herkogamy during the female phase*.* However, their gynophores bend to one side with the male phase and show lateral herkogamy*.* In this study, we observed the effect of successive sexual organs movement on variation in herkogamy traits. By artificially manipulating the flower to present gynophore straightened in the floral center or bend to one side, we attempted to investigate whether herkogamy movement affects pollinator access efficiency, pollen removal and deposition, and seed set ratio. Furthermore, we conducted artificial pollination in the female phase to evaluate the effect of changes in pollination environment on the variations in herkogamy traits. The results showed that gynophore straightened in female phase favors pollen deposition, whereas gynophore bending in male phase was conducive to the removal of pollen. Visitation frequency, pollen deposition and removal, and seed set ratio decreased significantly when the gynophore movement was manipulated. Finally, the bending of gynophore was obviously promoted by pollination. Therefore, the continuous variation of herkogamy in the same flower of *E. fischeriana* caused by the bending of the gynophore could improve the accuracy of pollination and avoid the interference of the ovary with access efficiency. That may be an adaptive strategy when pollinators are scarce. Furthermore, our study also provides good support for the hypothesis that variations in herkogamy traits are strongly selected by differences in pollination environments.

## Graphical abstract

In this study, we first reported the consecutive expression of two types of herkogamy in the same flower in Euphorbiaceae, and explored the effects of this continuous variation in herkogamy on its reproductive success. Euphorbia fischeriana, belonging to Euphorbiaceae, which have both vertical and lateral herkogamy, they show vertical herkogamy during the female phase, but their pedicels bend to one side at the male phase and show lateral herkogamy.

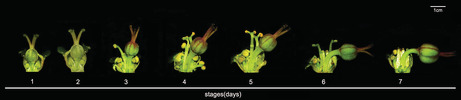

## 1 INTRODUCTION

Morphological or spatial–temporal variation in stigma or stamen movement is important in the sexual interference and reproduction of flowering plants (Abdusalam & Tan, 2014; Bynum & Smith, 2001; Dole, 1990; He et al., 2006; Lloyd, 1992; Wang et al., 2017; Ye et al., 2019). The degree of spatial and temporal separation of floral sexual organs (herkogamy and dichogamy, respectively) affects male and female fitness of bisexual flowers, prevents self‐pollination, and reduces interference between male and female organ functions (Barrett, 2002a; Leite et al., 2016; Ren & Tang, 2012; Wang et al., 2017). Herkogamy is mainly thought to reduce interference between male and female functions for plants with homostylous flowers (Barrett, 2002a, 2002b; Li et al., 2013; Lloyd & Webb, 1986; Webb & Lloyd, 1986). Nevertheless, any spatial separation between male and female organs has an associated problem of pollination inaccuracy (Lloyd & Webb, 1986). Floral mechanisms through interactions of herkogamy and dichogamy may decrease the interference between pollen removal and pollen receipt, thus maintaining pollination accuracy (Armbruster et al., 2014; Ganie et al., 2015; Medan & Ponessa, 2003). The fact that male and female activities occur at different times, and furthermore, that stamen and stigma successively occupy the same position for pollination at male and female phases, constitute an efficient floral system to address the herkogamy dilemma. This movement herkogamy mechanism could be accomplished by sequential stamen movement (Armbruster et al., 2014; Ren & Tang, 2012; Xiao et al., 2017), elongation of the style (Guo et al., 2014; Mamut et al., 2014), or exchange position of the stamen and stigma (Ye et al., 2019). Authors of these investigations found that continuous herkogamy successfully avoided sexual interference and maintained pollination precision. It is still unknown, however, how these movements influence the link between pollen removal and deposition by pollinators.

There are several types of herkogamy, such as vertical and lateral herkogamy. The most prevalent kind of herkogamy, vertical herkogamy, involves a vertical displacement between the stigma and anthers. Approach herkogamy occurs when the stigma is positioned above the anthers so that pollinators contact it before pollen; reverse herkogamy occurs when the stigma is positioned below the anthers so that pollinators contact it after pollen (Webb & Lloyd, 1986). Lateral herkogamy is a less common type of spatial separation between sexual organs in which the style is horizontally displaced from the center of the flower to create an angle with the stamens (Webb & Lloyd, 1986). Lateral herkogamy has been described in *Linum* (Ruiz‐Martín et al., 2018) and *Centaurium* (Brys & Jacquemyn, 2011; Ruiz‐Martín et al., 2018). Continuous herkogamy is a continuous variation encompassing different types of herkogamy (Forrest et al., 2011; Kulbaba & Worley, 2012). The majority of studies on continuous herkogamy are focused on the heterostyly, a reciprocal form of herkogamy in which there are two or three mutually different variants in the same breeding population (Barrett, 2002b; Barrett et al., 2000), as well as on other non‐reciprocal polymorphisms associated with heterostyly but at different stages in its evolution (e.g., Pérez‐Barrales et al., 2018; Pérez‐Barrales & Arroyo, 2010). Some plant species demonstrate continuous fluctuation in the separation of sex organs from reverse to approach herkogamy, which does not alter the growth of flowers, but reflects the selection of different pollinators (Forrest et al., 2011; Kulbaba & Worley, 2008, 2012, 2013). For example, in *Polemonium brandegeei*, a species pollinated by both hawkmoths and hummingbirds and with a continuous fluctuation from reverse to approach herkogamy, it has been shown that hawkmoths prefer reverse herkogamy, whereas hummingbirds prefer approach herkogamy (Kulbaba & Worley, 2012, 2013). Other studies have found that continuous variation in the same type of herkogamy at the individual level provides reproductive assurance to the plant. For example, in *Primula helleri*, herkogamy early in anthesis may enhance outcrossing potential, while its decrease later could enable reproductive assurance via delayed autonomous selfing in some but not all plants (de Vos et al., 2012). Contrastly, an uncommon phenomenon is the consecutive expression of two forms of herkogamy in the same flower. In *Lysimachia arvensis*, flowers exhibit lateral herkogamy on the first day of opening, but their styles later shift to a central position, exhibiting vertical herkogamy on the second day (Jiménez‐López et al., 2019). Some studies have shown that this consecutive variation has a high degree of heritability and is unlikely to be the result of developmental instability (Debat & David, 2001; Dongen, 2000; Jiménez‐López et al., 2019). Opedal (2018) has suggested that variation in herkogamy among populations and species represents adaptation of the mating system to variation in the local reproductive environment. Therefore, this consecutive expression of two forms of herkogamy at individual level may also be strongly selected by differences in pollination environments (Herlihy & Eckert, 2007; Jiménez‐López et al., 2019; Takebayashi et al., 2006). Experimental evidence, however, is scarce.

**FIGURE 1:**
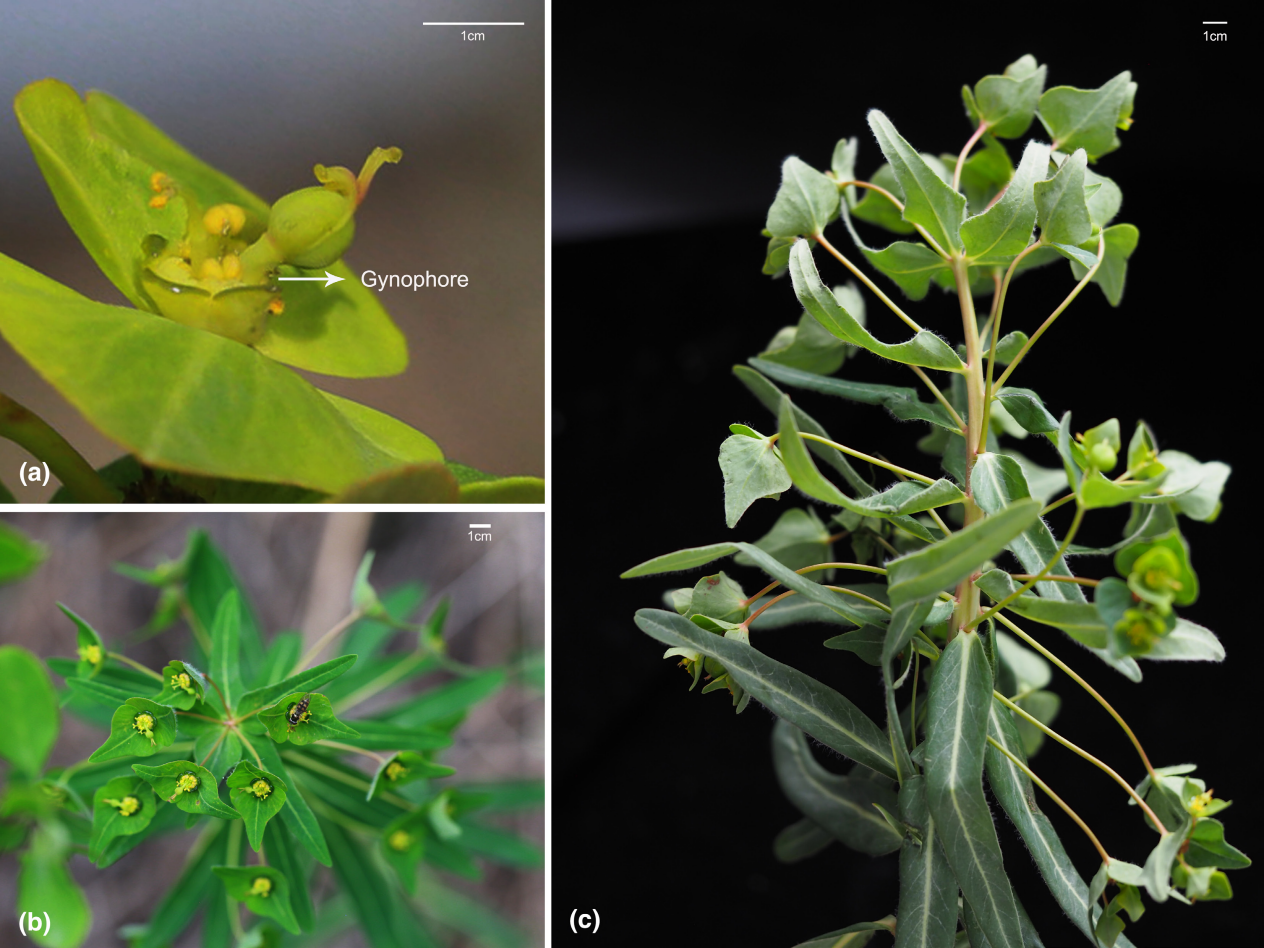
Floral morphology and inflorescence trait of *E. fischeriana*. (a) indicates the cyathia morphology; (b) to (c) show the inflorescence trait. Scale bar = 1 cm.

*Euphorbia fischeriana* Steud. is a Euphorbiaceae plant with reduced unisexual flowers arranged into distinctive pseudanthia called cyathia. Each cyathium is solitary at the apex of the dichotomous branch and consists of a cup‐shaped involucre with four glands on its rim, which encloses many male flowers and a single pistillate flower in its center (Figure 1a,b). A *E. fischeriana* cyathium has both vertical and lateral herkogamy, which exhibits vertical herkogamy during their female phase (female flowers mature and male flowers are undeveloped), but their gynophores bend to one side at the male phase (male flowers mature while female flowers wilt) and show lateral herkogamy. Considering that a typical cyathium with both staminate and pistillate flowers resembles a bisexual flower functionally (Rabot & Hayden, 2017; Figure 1a), we use  the term flower to refer to the cyathium. Therefore, we speculate that the shift during anthesis from vertical to lateral herkogamy affects the deposition and removal of pollen in the different flowering stages, and might be influenced by the pollination environment. To test this hypothesis, we aimed to study if changes in herkogamy features impact pollen removal, deposition, and pollinator efficiency by artificially manipulating the flower to show pistils in the floral center or bend to one side. Furthermore, artificial pollination was used to assess the influence of pollination environment alteration on herkogamy movement. On the other hand, to determine whether herkogamy traits were constant within plants, it is necessary to measure the correlation between herkogamy values obtained from each plant, as well as to characterize other correlations between floral traits. Specifically, our two objectives were to examine: (i) whether and how continuous herkogamy promote pollen output and deposition; (ii) whether and how changes in pollination environment influence continuous herkogamy.

## 2 MATERIALS AND METHODS

### 2.1 Study species and sites

*Euphorbia fischeriana* Steud. is a perennial flowering plant, which grows in grasslands and hillsides at an altitude of 200–3300 m (Ma & Michael, 2008). The Cyathium is sessile with campanulate involucre and four glands. The ovary of the female flower is pedicellate and exsert from the cup (Ma & Michael, 2008). Functionally, the cyathium inflorescence is analogous to a single flower (Figure 1a). Therefore, we use the gynophore and the flower to represent the pedicel of the female flower and the cyathia (Figure 1a). We conducted field investigations from May to July 2021 in a natural population located in Xinglongshan National Natural Reserve (35°55′00″ N; 103°50′50″ E, alt. 1990 m), Lan Zhou, China. This population contained more than 1000 individuals of *E. fischeriana*. In this sample plot, the most common plants are *Vicia tetrasperma, Oxytropis xinglongshanica*, *Astragalus mahoschanicus, Pedicularis sfriata,* and *Artemisia sacrorum*. The average annual temperature is 7.8°C, and the average annual rainfall is 380 mm. The temperature range during the sampling period (July) was 4–29°C, and the rainfall was 43.0 mm.

### 2.2 Floral characteristics, pollen viability, and stigma receptivity during flower development

**FIGURE 2:**
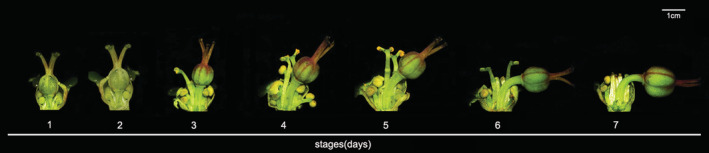
Floral development and variation in herkogamy traits of *E. fischeriana* (the number indicates the time after flower opening (days)). Scale bar = 1 cm.

To monitor the changes in floral characteristics and herkogamy during flowering, 30 randomly selected buds from 10 separate plants were tagged and the flowering process of each flower was recorded after it bloomed. The changes in flower characters, especially the position of pistil and stamens, were recorded at 9 a.m. every day during the whole flowering period. According to the relative position of the stigma and stamens, the floral development was divided into three phases: female phase (1–3 days after flower opening), the stigma is mature, and the gynophore is upright and located above the stamens (vertical herkogamy); middle phase (4–5 days after flower opening), stamens begin to mature while the gynophore is not completely curved (lateral herkogamy); male phase (6–7 days after flower opening), the anthers mature and the stigmas are completely bent to one side (lateral herkogamy) (Figure 2).

At the same time as floral characteristics and herkogamy measuring, the changes in pollen viability and stigma receptivity during flower development were also measured. A total of 210 flower buds from 21 individuals were selected and bagged. Pollen viability and stigma receptivity of 30 marked flowers were measured every 24 h for 7 days. The acceptability of stigma was tested by benzidine–hydrogen peroxide method. The pollen germination rate in a sucrose solution with a 10% by‐weight concentration was used to determine pollen viability (Dafni et al., 2005; Duan et al., 2005).

### 2.3 Pollinator observations and mating system

In this part of the study, we focused on visitor insects to understand whether different floral phases (different herkogamy types) affected the behavior of visitors. To test whether different floral phases led to alter behavior of visitors, we randomly selected 20 individual plants to conduct visitor observations at the same time on 5 sunny days (from 9:00 a.m. to 5:00 p.m.). We recorded whether each flower is in the male phase or the female phase every day. During the surveys, we observed and recorded the number of visit times of the different floral phases (including female, middle, and male phases) of flowers and the foraging behavior of visitor insects, every flowering phase for a minimum of 45 hours was observed, and the total observation duration exceeded 135 hours. Insect specimens were collected in specimen boxes for later identification. Duan et al. (2005) observation methods were employed to record visitor species, visitor behavior, and visitation frequency.

Furthermore, the breeding system of *E. fischeriana* was also investigated. A total of 120 flower buds from 12 separate plants were randomly tagged and bagged. Then, four treatments were conducted when flowers opened (*N* = 30 respectively): (1) self‐pollination in which stigma were pollinated by pollen of the same flower (self‐pollination carried out on the 4th day (middle phase of *E. fischeriana*)); (2) cross‐pollination in which stigma were pollinated by pollen of the other plant from at least 10 m away; (3) natural pollination; (4) bagged in which flowers were bagged before blossoming until harvest. Furthermore, all the seeds were collected when mature, and the seed set ratio was determined by dividing the number of plump seeds by the total number of ovules per fruit.

### 2.4 Correlation between sex organ growth and variation in herkogamy traits

We randomly collected between 200 and 300 plants and measured herkogamy traits for two flowers per plant, and herkogamy characteristics were measured in the field with vernier calipers and protractors (*N* = 180). The angle between the gynophore and the stamens (hereafter “gynophore‐stamen angle”) was used to calculate lateral herkogamy. We measured stamen length (from flower base to anther center; stamen artificially straightened) and pistil length (from flower base to stigma center; pistil artificially straightened) to characterize vertical herkogamy; vertical herkogamy was then calculated as the difference between pistil and stamen lengths, which would be equivalent to final separation between anthers and stigma (hereafter “stigma‐anther displacement”). For the flowers measured in the field, correlations between pistil and stamen lengths, stigma–anther displacement, and gynophore–stamen angle were determined.

### 2.5 Effects of artificial interruption of continuous herkogamy variation on visitation frequency, pollen removal, deposition, and seed set ratio

To artificially interrupt the continuous herkogamy variation in *E. fischeriana*, the floral carpophore was delicately leaned, and the gynophore was immobilized with a white thread to prevent further deflexion. According to the manipulations conducted by Ren and Bu (2014), one end of the thread was attached to the floral carpophore and the other end was attached to the gynophore through the area between two opposed petals. We disguised the threads to keep them out of the route of pollinators in order to reduce potential disturbance during insect visits. In addition, considering that the stamens of *E. fischeriana* are small and do not bend, we only conducted artificial control experiments on the pistils.

**FIGURE 3:**
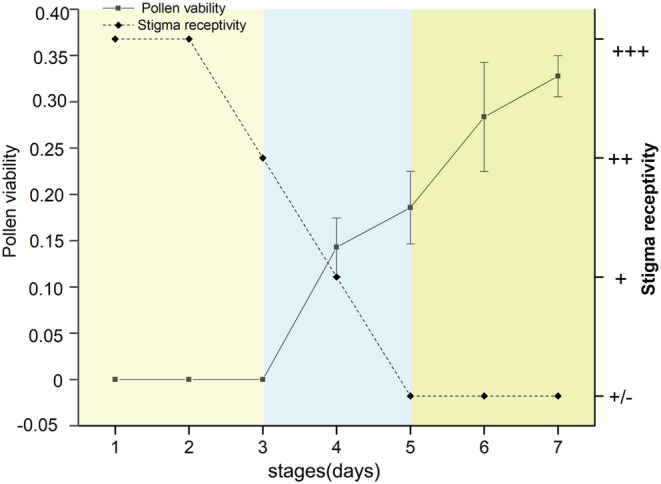
The pollen viability (± s.d.) and stigma receptivity on different days in *E. fischeriana*. Indicating pollen viability (solid line) and stigma receptivity (dotted line). The dynamics of stigma receptivity “−/+” means that some of the stigmas are receptive, “+” means stigma is receptive, and the more “+” means stigma is strongly receptive.

**FIGURE 4:**
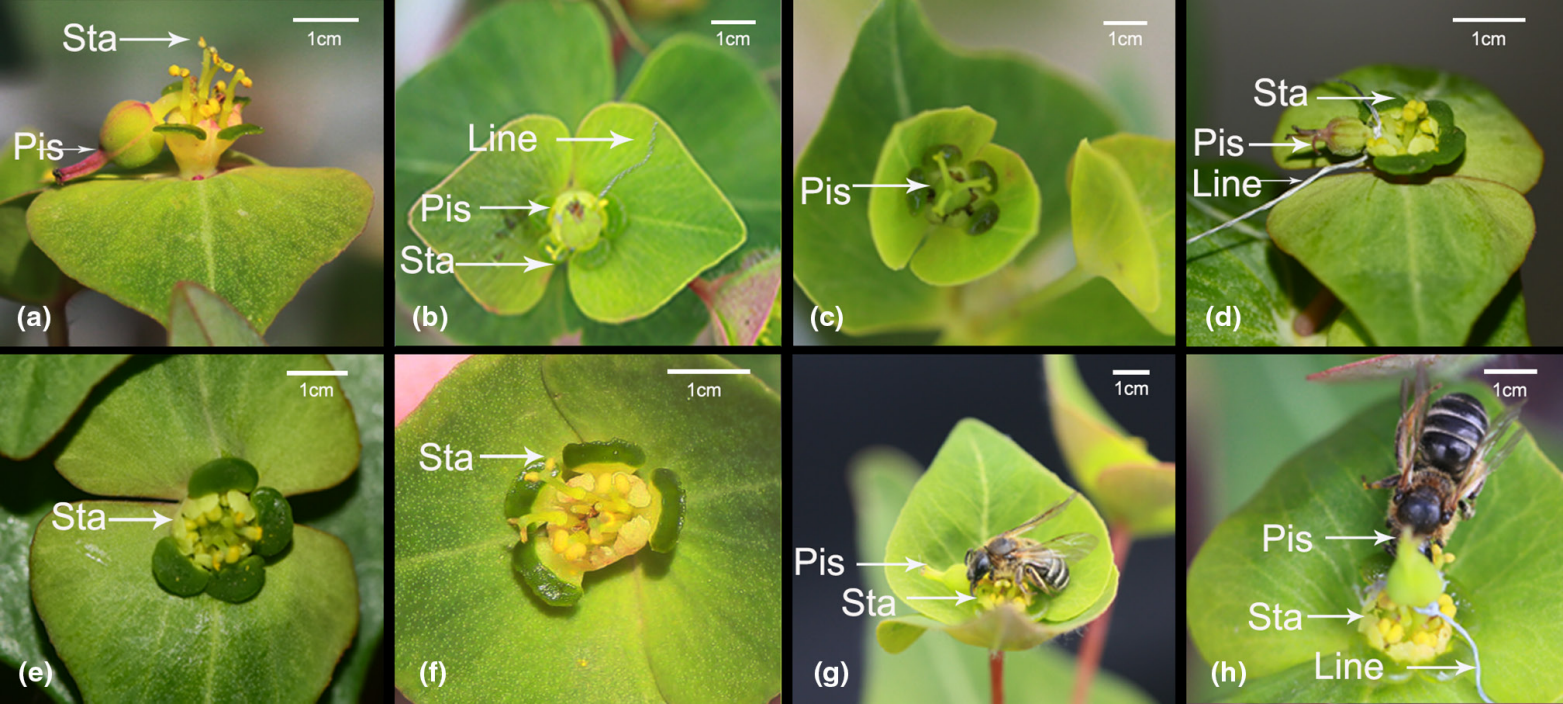
Pollinators and experimental treatment of *E. fischeriana*. (a) In nature, an *E. fischeriana* flower in the male phase (lateral herkogamy); (b) artificial treatment imitates vertical herkogamy in the male phase; (c) in nature, an *E. fischeriana* flower in the female phase (vertical herkogamy); (d) artificial treatment imitates lateral herkogamy in the female phase; (e) artificial treatment that an *E. fischeriana* flower removed pistil in the female phase; (f) artificial treatment that an *E. fischeriana* flower removed pistil in the male phase; (g) vertical herkogamy flower being visited by *A. carbonaria*; and (h) lateral herkogamy flower being visited by *A. carbonaria*; “Sta, stamen” and “Pis, pistil”. Scale bar = 1 cm.

**FIGURE 5:**
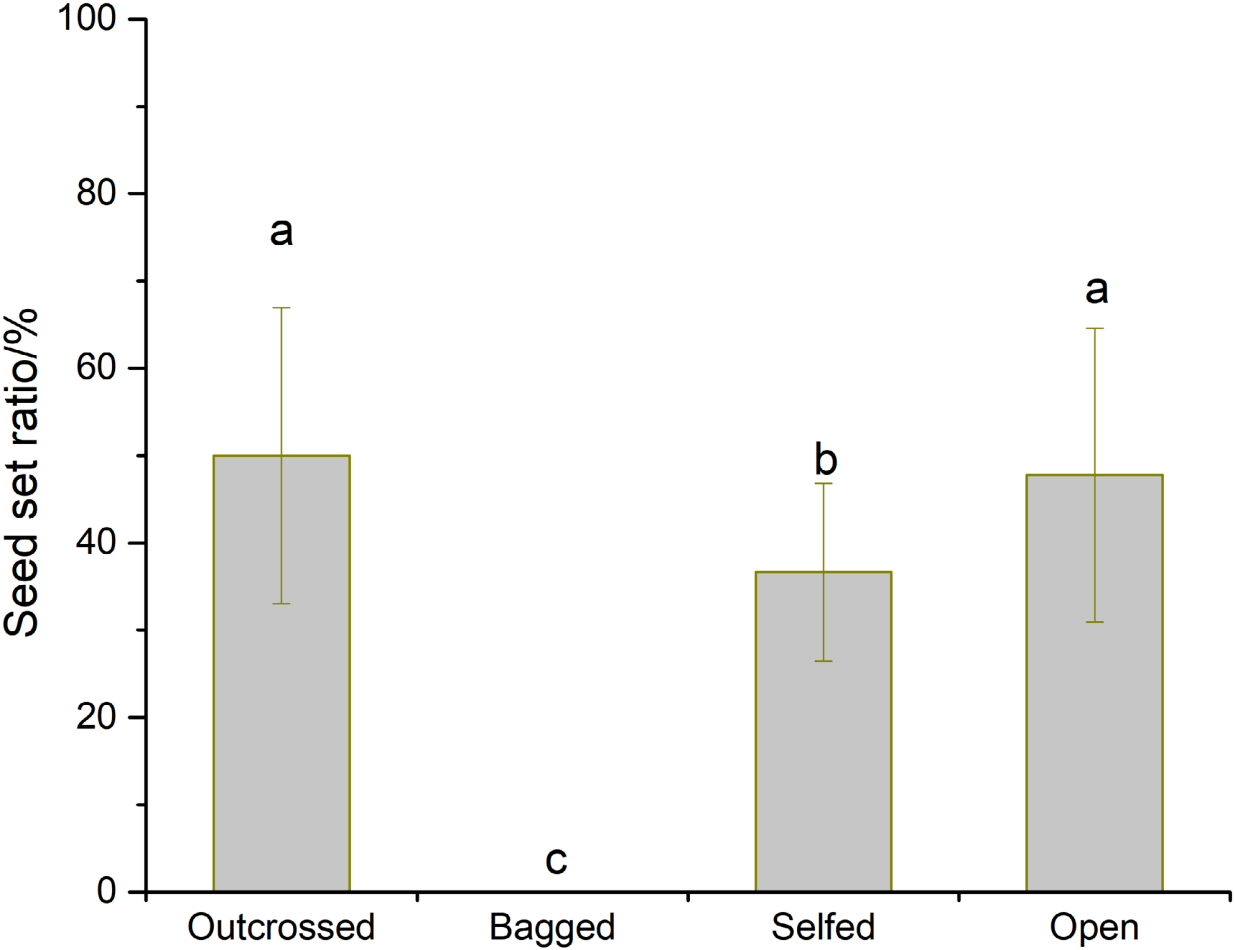
Breeding system of *E. fischeriana*. Bagged (± s.d.), bagged without any treatment (± s.d.); Selfed (± s.d.), self‐pollination; Open (± s.d.), natural pollination (± s.d.); Outcrossed (± s.d.), outcrossed pollination. Different letters on items indicate significant differences at the 0.05 level.

In order to test the effect of interruption of herkogamy movement on pollinators, we randomly selected 120 flowers (60 flowers in male phase and 60 flowers in female phase) from 20 plants for the following treatments: (1) vertical gynophore in female phase (vertical herkogamy, *N* = 20): selected 20 flowers under normal conditions in female phase (Figure 4c); (2) gynophore bending in female phase (lateral herkogamy, *N* = 20): selected 20 flowers after artificial gynophore bending in female phase (Figure 4d); (3) removing pistil in female phase (*N* = 20): selected 20 flowers after removing pistil in female phase (Figure 4e); (4) vertical gynophore in male phase (vertical herkogamy, *N* = 20): selected 20 flowers after gynophore is artificially erecting in male phase (Figure 4b); (5) gynophore bending in male stage (lateral herkogamy; *N* = 20): selected 20 flowers under normal conditions in male phase (Figure 4a); and (6) pistil removal in male phase (*N* = 20): selected 20 flowers after pistil removal in male phase (Figure 4f). After manipulation, we conducted visitor observations in each of the experimental groups between 09:00 a.m. and 5:00 p.m. at least 45 h, respectively, and the total observation time was more than 270 h, and then the visitation frequency and the foraging behavior of visiting species were recorded.

To examine the effect of interrupted herkogamy movement on pollen removal and deposition, we randomly labeled 80 flower buds on 30 plants for bagging, and each flower was randomly assigned to the following treatment after flowering: (1) vertical gynophore in female phase (vertical herkogamy, *N* = 20) (Figure 4c); (2) gynophore artificially bending in female phase (lateral herkogamy, *N* = 20) (Figure 4d); (3) gynophore artificially erecting in male phase (vertical herkogamy, *N* = 20) (Figure 4b); and (4) gynophore bending in male stage (lateral herkogamy, *N* = 20) (Figure 4a). For treatments 1 and 2, after treatment, flowers are exposed to pollinators in female phase. The flowers were removed after they had received a visit and stored separately in vials containing 70% ethanol. To assess pollen deposition per visit, the stigmas were cleared for 30 min in 1 mol/L NaOH, squashed, and examined under light microscopy (4×). Then, for treatments 3 and 4, after treatment, flowers are exposed to pollinators in male phase. The flowers were removed after they had received a visit and stored separately in vials containing 70% ethanol. We randomly collected 20 undehisced flowers from different plants to assess the mean pollen production per anther. Pollen grains were detached from the dehisced anthers and counted in five 20‐lL subsamples from each flower under light microscopy (4×). We then divided the total number of pollen grains remaining by the number of dehisced anthers on the flower to estimate the mean number of pollen grains remaining per anther. Pollen removal per visit was calculated by subtracting the number of pollen grains remaining per anther from the mean pollen production per anther.

To test the effect of the interruption of herkogamy movement on the reproductive success of *E. fischeriana*, we randomly selected 60 flower buds from 30 plants and bagged them. After flowering, the following treatments were carried out: (1) vertical gynophore during the whole flower period (vertical herkogamy, *N* = 20); (2) gynophore bending during the whole flower period (lateral herkogamy, *N* = 20); and (3) natural control without any treatment. After manipulation, these flowers were exposed to pollinators (*N* = 20). We made a special note of whether each visitor was able to touch the stigma and dehiscing anthers. All anthers were harvested after 7 days and utilized to calculate the pollen export percentage. The underdeveloped ovules and seeds from the remaining 40 modified and 20 control flowers were counted 2 weeks later, and the seed set ratio was determined by dividing the number of plump seeds by the total number of ovules per fruit.

### 2.6 Effects of pollination environment change on herkogamy movement

To detect the effects of pollination environment changes on herkogamy movement, we bagged 60 flower buds from 20 plants. Three treatments (*N* = 20 respectively) were conducted at female phases of anthesis: (1) hand self‐pollination, in which flowers were manually pollinated by their own pollen grains; (2) hand cross‐pollination, in which flowers are emasculated and manually pollinated with pollen grains from other individuals up to 100 m away; and (3) bagged, in which flowers are bagged before blossoming to minimize the impact of outsiders. From opening to withering, the state of flower, especially the position of stigma and stamens, the state of anthers dehiscence, and the state of stigma maturity, were monitored twice a day. The angle of gynophore bending was recorded every 2 h until it was completely bent (90°). We calculated the bending rate: bending rate = the bending angle /the bending time, and bending proportio*N* = the bending number/total samples.

### 2.7 Data analysis

All values are presented as mean (± standard deviation, SD). One‐way analysis of variance (ANOVA), with the post hoc Tukey test as the multiple pairwise comparison test, was used to determine any significant differences (*p* < .05) in pollen viability, pollen export, pollen deposition, seed set ratio, visiting frequency, and bending rate under different treatments. Variation in herkogamy traits among plants was tested by means of general linear models (GLMs). Pearson correlations between pistil and stamen lengths, herkogamy values, stigma–anther displacement, and gynophore–stamen angle were calculated for the flowers measured in the field. The SPSS 22.0 statistical software package was used to calculate and analyze the comparative test results. All graphs were constructed using Origin 9.1 software (OriginLab, Northampton, MA, USA).

## 3 RESULTS

### 3.1 Floral characteristics, pollen viability, and stigma receptivity during flower development

In natural conditions, the gynophore of *E. fischeriana* is mature and erect in the center of the flower when flowers open. At this stage, the anther is significantly lower than the stigma, showing vertical herkogamy (Figures 2, 3). On the third day of flowering, the gynophore begins to bend to one side and the receptivity of stigma begins to decline. At this time, the gynophore and anther are at a certain angle, showing lateral herkogamy (Figures 2, 3). With the further bending of gynophore, anthers began to mature on the 4th day. When the gynophore was completely bent to one side, the stigma begins to wilt. At this time, the pollen activity increased further and reached its peak on the 7th day of flowering (Figures 2, 3).

### 3.2 Pollinator observations and mating system

For our observation, *E. fischeriana* has three different insect species, including *Andrena carbonaria*, *Euaspis basalis*, and *Mimesa equestris* (Table 1, Figure 4). Only *A. carbonaria*, one of the three species of insects present, could successfully deposit pollen grains on the stigma. The stigma made contact with the pollinator's head and was deposited with pollen grains when the pollinator visited the flower during the female phases (vertical herkogamy), while the dehisced anthers were placed outside of the bottom. Dehiscing anthers discharged pollen grains on the front of the pollinator's head during the male phase (lateral herkogamy), but the non‐receptive stigma was situated away from the spot where the pollinator anthers made contact (Figure 4).

**TABLE 1:** Visiting frequency and taxa of pollinators at different flowering phases in *E. fischeriana*.

Frequency of visits (visitation frequency/3 h)			
Species	Female	Middle	Male
*Andrena carbonaria*	0.071 ± 0.023^c^	0.031 ± 0.016^b^	0.153 ± 0.025^a^
*Euaspis basalis*	0.002 ± 0.006^b^	0^b^	0.016 ± 0.013^a^
*Mimesa equestris*	0^b^	0.005 ± 0.010^b^	0.011 ± 0.012^a^

*Note*: Values are mean ± SD. Different letters on items indicate significant differences among flowering phases at the 0.05 level.

Our results suggest that there is no pollen limitation for *E. fischeriana* in natural conditions as the seed set ratio of the outcross pollination treatment (50.000% ± 16.986) was not significantly different from the natural condition (47.773% ± 16.834, *F*_3,116_ = 95.186, *P* = 0.508). Furthermore, *E. fischeriana* could not be seeded after bagging, indicating its inability to undergo autonomous selfing, whereas its propagation depended on pollinators. Moreover, artificial selfing significantly reduced the seed set ratio (36.640% ± 10.191, *F*_3,116_ = 95.186, *p* < .0001; Figure 5), indicating that certain inbreeding depression occurs in *E. fischeriana*.

### 3.3 Correlation between sex organ growth and variation in herkogamy traits

**FIGURE 6:**
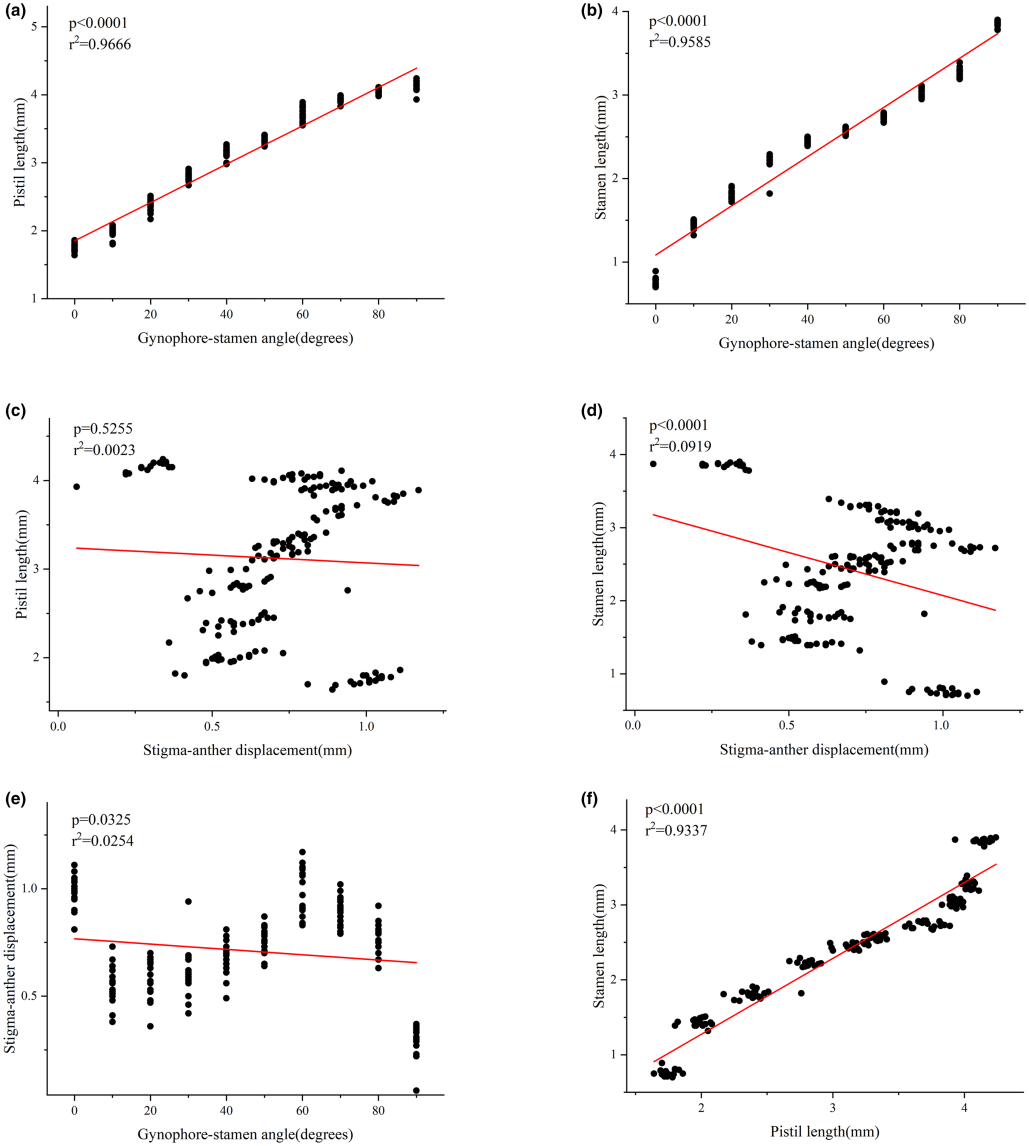
Pearson correlations between floral traits of *E. fischeriana*. (a) Pistil length/gynophore–stamen angle, (b) stamen length/gynophore–stamen angle, (c) pistil length/stigma–anther displacement, (d) stamen length/stigma–anther displacement, (e) gynophore–stamen angle/stigma–anther displacement, and (f) Pistil/stamen length. Sample sizes are 180 in all cases.

In plants measured in the field, pistil length ranged from 1.64 to 4.24 mm (3.12 mm ± 0.83, *N* = 180) and stamen length was between 0.70 and 3.90 mm (2.41 mm ± 0.87, *N* = 180). Gynophore–stamen angle ranged from 0 to 90°, whereas stigma–anther displacement ranged from 0.06 to 1.17 mm with a mean of 0.71 ± 0.22 mm (*N* = 180). There is a positive correlation between the length of the sex organs, that is, with the elongation of the pistil, the stamen is also growing (GLM, estimate: 1.013, *t* = 50.067, *df* = 178, *p* < .0001; Figure 6f). Furthermore, gynophore–stamen angle (lateral herkogamy) was significantly correlated with the other three traits (Figure 6a,b,e); gynophore–stamen angle was positively correlated with both pistil (GLM, estimate: 0.028, *t* = 71.760, *df* = 178, *p* < .0001) and stamen length (GLM, estimate: 0.030, *t* = 64.122, *df* = 178, *p* < .0001), but negatively correlated with stigma–anther displacement (GLM, estimate: −0.001, *t* = −2.155, *df* = 178, *p* = .0325). In contrast, stigma–anther displacement (vertical herkogamy) was negatively correlated with the stamen length (GLM, estimate: −1.176, *t* = −4.245, *df* = 178, *p* < .0001) but not with pistil length (GLM, estimate: −0.176, *t* = −0.636, *df* = 178, *p* = .5255), and the vertical distance between stigma and anther decreases significantly with the growth of stamen (Figure 6c,d).

### 3.4 Effects of artificial interruption of continuous herkogamy variation on visitation frequency, pollen removal, deposition, and seed set ratio

In female‐phase flowers, compared with the natural state (VH, 0.082 ± 0.018), artificial gynophore removal (RP, 0.156 ± 0.019) and gynophore bent (LH, 0.142 ± 0.017, *F*_5,84_ = 270.447, *p* < .0001) increased visitation frequency, while there was no significant change in the visitation frequency compared to the gynophore removed and gynophore‐bent treatment. In male‐phase flowers, compared with the natural state (LH, 0.289 ± 0.023) and artificial gynophore removal (RP, 0.333 ± 0.040), gynophore erect (VH, 0.111 ± 0.019, *F*_5,84_ = 270.447, *p* < .0001; Table 2) decreased visitation frequency, while visitation frequency of insects to the gynophore removed flowers was higher than that to the gynophore‐erect flower.

**TABLE 2:** Effects of artificial interruption of continuous herkogamy variation on visitation frequency in *E. fischeriana*.

Species	Frequency of visits (visitation frequency/3 h)					
	Female phase			Male phase		
	VH	LH	RP	VH	LH	RP
*Andrena carbonaria*	0.082 ± 0.018 ^e^	0.142 ± 0.017^d^	0.156 ± 0.019^d^	0.111 ± 0.019^c^	0.289 ± 0.023^b^	0.333 ± 0.040^a^
*Euaspis basalis*	0.027 ± 0.012^b^	0.042 ± 0.015^a^	0.040 ± 0.009^a^	0.029 ± 0.012^b^	0.040 ± 0.018^a^	0.041 ± 0.018^a^

*Note*: Values are mean ± SD. Different letters on items indicate significant differences among treatments at the 0.05 level.

Abbreviations: LH, lateral herkogamy; RP, remove pistil; VH, vertical herkogamy.

**FIGURE 7:**
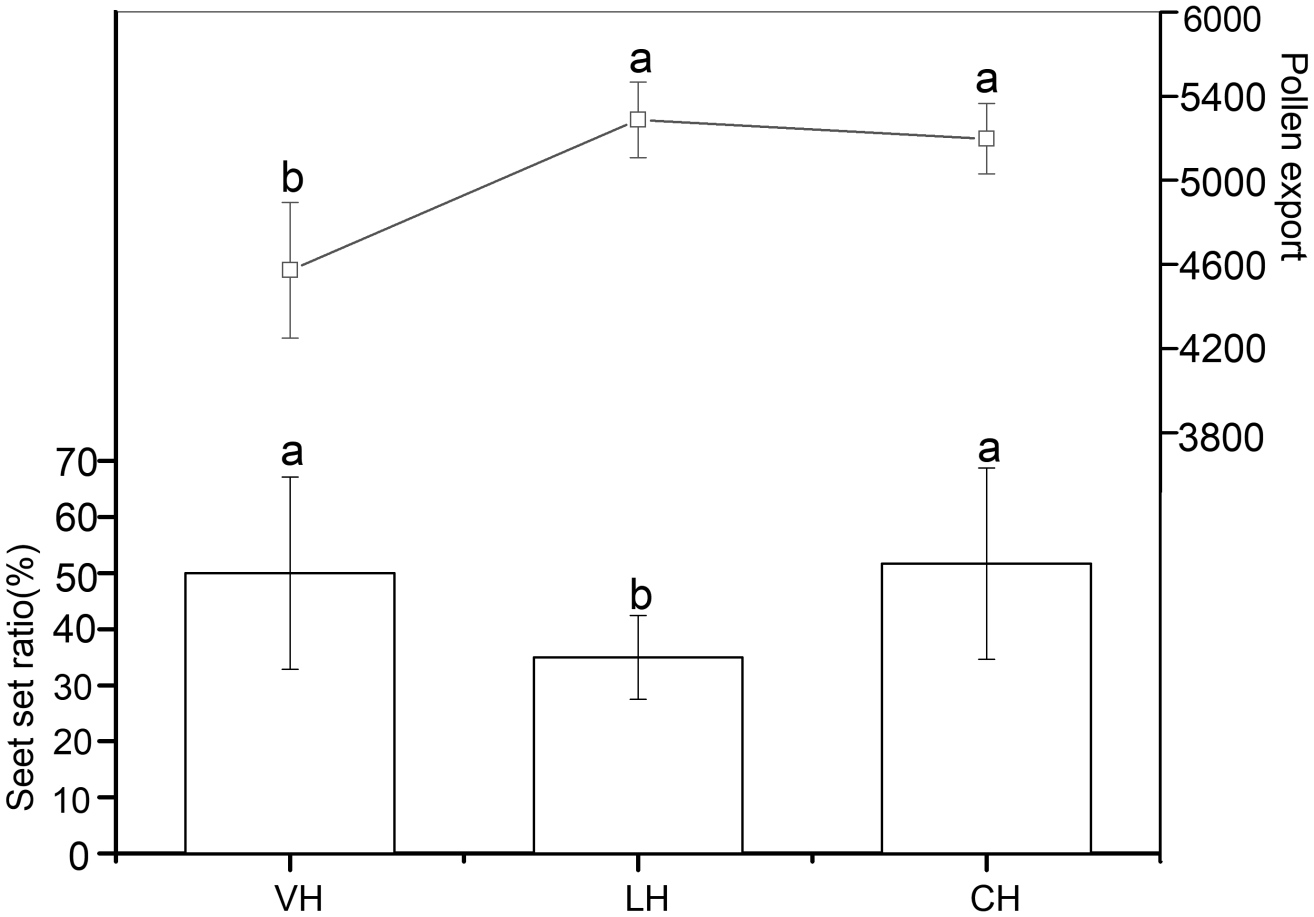
The effect of different types of herkogamy treatments on pollen export (± s.d.) and seed set ratio (± s.d.) of *E. fischeriana*. VH vertical herkogamy; LH lateral herkogamy; CH Continuous herkogamy. Different letters on items indicate significant differences at the 0.05 level.

Compared with the natural condition (35.400 ± 8.519), artificial bending of the gynophore (5.550 ± 8.720, *F*_*1*,38_ = 119.907, *p* < .0001; Table 3) in the female phase significantly reduced the pollen deposition of *E. fischeriana*, while in the male phase, the pollen removal number of the upright gynophore treatment (VH, 770.000 ± 178.001) was significantly lower than that of the natural control treatment (LH, 1638.000 ± 258.713, *F*_1,38_ = 152.798, *p* < .0001; Table 3). In addition, our results showed that artificially keeping the gynophore (lateral herkogamy) bent throughout the flowering period significantly reduced the seed set ratio of *E. fischeriana* (50.000% ± 17.134, 34.970% ± 7.469, and 51.670% ± 17.048 for vertical herkogamy, lateral herkogamy, and continuous herkogamy, respectively; *F*_2,57_ = 7.931, *p* = .001; Figure 7). On the contrary, artificial erect gynophores (vertical herkogamy) significantly reduced pollen removal number (4772.500 ± 323.010, 5487.500 ± 179.820, and 5397.500 ± 168.175 for vertical herkogamy, lateral herkogamy, and continuous herkogamy, respectively; *F*_2,57_ = 55.164, *p* < .0001; Figure 7). The above results indicate that the continuous variation in herkogamy in *E. fischeriana* plays an important role in improving the efficiency of pollen removal and deposition.

**TABLE 3:** Effects of artificial interruption of continuous herkogamy variation on pollen removal and deposition in *E. fischeriana*.

	Removal (male phase)	Deposition (female phase)
VH	770 ± 178^b^	35.4 ± 8.52^a^
LH	1638 ± 258.71^a^	5.55 ± 8.72^b^

*Note*: Values are mean ± SD. Different letters on items indicate significant differences between treatments at the 0.05 level.

Abbreviations: LH lateral herkogamy; VH vertical herkogamy.

### 3.5 Effects of pollination environment change on herkogamy movement

**FIGURE 8:**
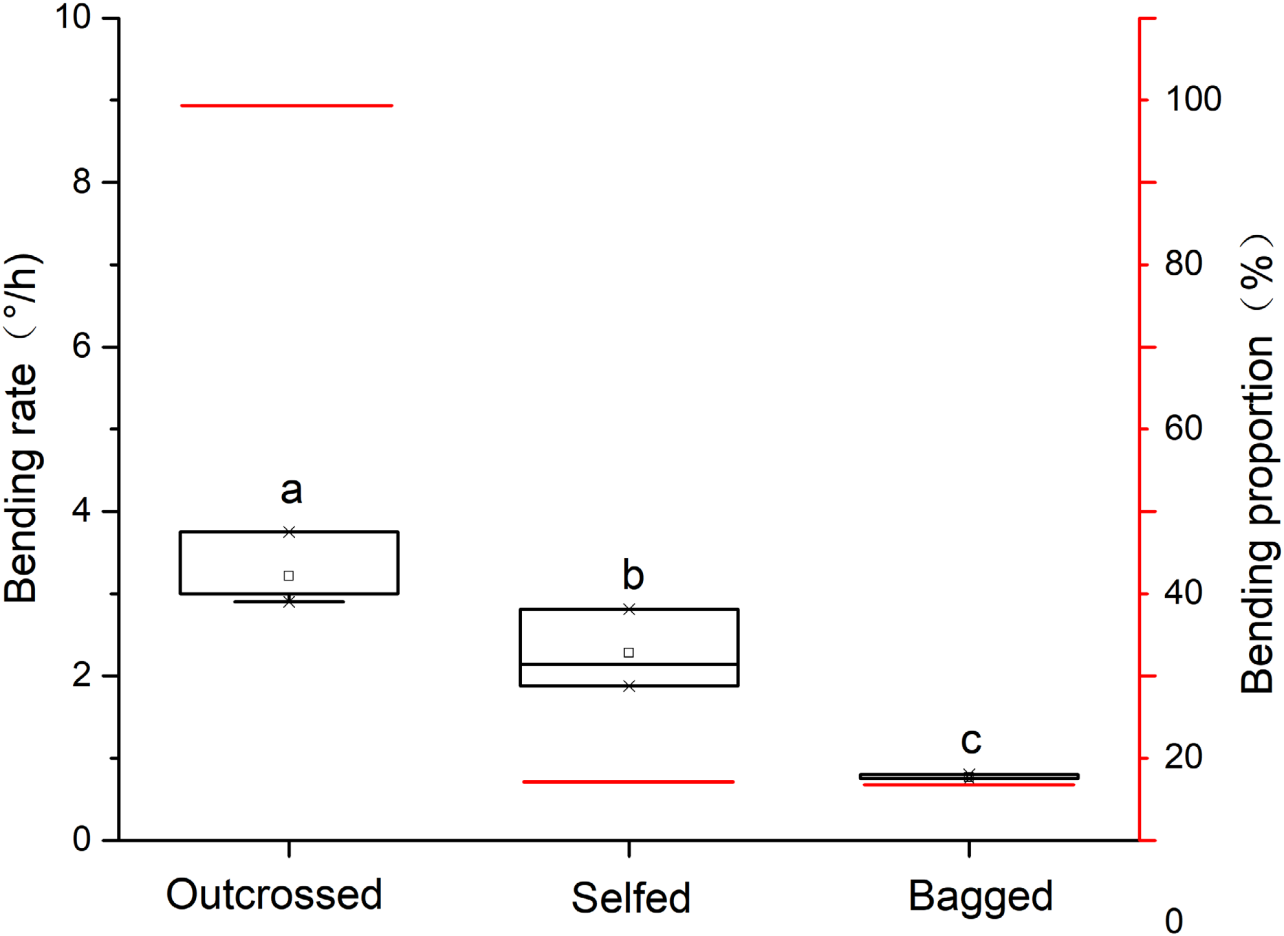
Effects of pollination environment changes on herkogamy movement. Bagged (± s.d.), bagged without any treatment; Selfed (± s.d.), self‐pollination; Outcrossed (± s.d.), outcrossed pollination. White boxplots indicate the bending rate on any treatment, showing medians, quartiles, interquartile ranges, and outliers. Red line indicates the bending proportion on any treatment. Different letters on items indicate significant differences at the 0.05 level.

Compared with the control condition (0.767°/h ± 0.029, 15%), artificial pollination (3.272°/h ± 0.349, 2.277°/h ± 0.480, *F*_2,23_ = 72.608, *p* < .0001) could significantly increase the bending rate of gynophore of *E. fischeriana*, and the bending rate of outcross pollination group (3.272°/h ± 0.349) was significantly higher than that of selfing group (2.277°/h ± 0.480, *F*_2,23_ = 72.608, *p* < .0001; Figure 8). However, self‐pollination does not change the bending proportion of gynophore (15%), but outcross pollination can significantly increase the bending proportion (100%; Figure 8), indicating that artificial pollination will affect the bending of gynophore of *E. fischeriana*, thus changing the movement of herkogamy.

## 4 DISCUSSION

Our observations on the flowering process of *E. fischeriana* showed that there are two types of herkogamy. In the female phase, the herkogamy shows vertical herkogamy, and in the male phase, the gynophore bends to one side, showing lateral herkogamy (Figure 2). In flowering plants, it is an unusual tactic (Jiménez‐López et al., 2019). In *E. fischeriana*, the lengths of the pistil and stamens were closely connected (i.e., flowers with longer pistils also had longer stamens). Like *Mimulus* (van Kleunen & Ritland, 2004), *Aquilegia* (Herlihy & Eckert, 2007), *Polemonium* (Kulbaba & Worley, 2008), and other species exhibiting stigma–height dimorphism, this taxon exhibits continuous variation in vertical herkogamy (such as Barrett et al., 2000; Barrett, 2019). In *E. fischeriana,* male and female sex organs show a different relationship with vertical herkogamy, and variation in the stigma–anther displacement depended mainly on variation in stamen length and not on the change in pistil (Figure 6). That is, stamen length variation mostly causes vertical herkogamy. In contrast to vertical herkogamy, both sexual organs correlated positively with lateral herkogamy. As in *L. arvensis*, the gynophore–stamen angle also depended mainly on the lengths of the sexual organs (Jiménez‐López et al., 2019). With the growth of pistils and stamens, the stigma–anther displacement decreased and gynophore–stamen angle increased, indicating that the transition into lateral herkogamy in the later stage is of great significance for reducing sexual interference and selfing in *E. fischeriana*.

Plants with a dichogamy mechanism may overcome the herkogamy dilemma by locating the stamen and stigma in the same location but at distinct periods of sexual activity (Armbruster et al., 2014; Li et al., 2001; Ye et al., 2019; Yu & Huang, 2006). The interactions of dynamic herkogamy and dichogamy not only completely avoided sexual interference but also ideally maintained pollination accuracy (Armbruster et al., 2014). For example, the accuracy of pollination and avoiding sexual interference can be achieved by exchanging the locations of stigma and stamen in *Ajuga decumbens* (Ye et al., 2019). Similarly, *Parnassia epunctulata* and *P. wightiana* resolve the herkogamy dilemma by accurate repositioning of stamens and stigmas (Armbruster et al., 2014; Xiao et al., 2017). A dynamic movement of gynophore has been discovered to perform several roles in plant reproduction, such as improving pollination in columbine (Yu & Huang, 2006), reducing interference between the reproductive functions of female and male organs in *Eremurus himalaicus* (Verma et al., 2004) and forming an assortative mating system in gingers (Li et al., 2001). In *E. fischeriana*, stigma matured first. With the flower development, the gynophore was bent to one side, and the anther became dehisce. Compared to the female phase, the anther occupies the original position of stigma in the male phase, resulting in a dynamic herkogamy with diverse sexual roles in the floral phase (Figures 2, 3). The insects visiting the flowers of *E. fischeriana* were a minority and few, only *A. carbonaria* could successfully deposit pollen grains on the stigma. The pollinator's head contacts the stigma during the female phases (vertical herkogamy) when it visits a flower, thus completing pollen deposition, at which point the anther contacts other parts of the pollinator's body. In contrast, at the male phases (lateral herkogamy), anthers and stigma exchange sites, and dehisced anthers release pollen grains at the pollinator head. Therefore, the anthers of the male phase and stigma of the female phase were in touch with the same spot on the body of the pollinator. As a result of the floral mechanism, the dehiscing anthers and receptive stigma appeared at the proper position and at the right time (Armbruster et al., 2014). Previous studies have shown that the straightened style during male phase might interfere with pollen shedding in *Chamerion conspersum* (Guo et al., 2014). A similar phenomenon was also found in *E. fischeriana,* straightened gynophore during male phase significantly reduced pollen removal. In addition, we found that forced gynophore bending during the female phase also resulted in a significant decrease in pollen deposition, which may be due to the inability of the curved stigma to effectively combine with the pollen stuck on the insect (Table 3). It is generally believed that the reduced efficiency of pollen transfer is due to the different contact sites of anther and stigma with insects, and our findings also support this hypothesis. Additional studies also corroborate that a single floral movement could have more than one adaptive significance (Buttrose et al., 1977; Schlessman, 1986). For example, stylar movement in *Eremurus himalaicus* (Liliaceae) avoids self‐pollination and promotes outcross pollination (Verma et al., 2004), which also ensures that the style is separated from the flight path of visitors. This isolation may avoid the effect of the pistil on pollen export and improve male fitness. In *E. fischeriana,* manipulation experiments showed that straightened gynophore during male phase significantly reduced the pollen removal and the visitation frequency (Tables 2, 3). These results demonstrated that gynophore movement in *E. fischeriana* reflects an adaptation to decrease the interference between female and male organs. Interestingly, however, the straightened gynophore of *E. fischeriana* during the whole flower period would significantly reduce the visitation frequency. In contrast, removing pistils during the whole period will increase the visitation frequency, and the same pattern was presented after bending the gynophore (Table 3). It is uncommon as previous studies have suggested that removal of sexual organs, such as emasculation, might decrease attraction to pollinators (Eckert et al., 2006; Schoen & Lloyd, 1992). This may be due to *E. fischeriana* offering nectar as a reward to pollinators, and the larger ovary will block the nectary gland below, thus reducing the attractiveness to pollinators. Therefore, *E. fischeriana* could present vertical herkogamy at the female phase to improve pollination accuracy, while transitioning to lateral herkogamy at the male phase to avoid interference of the ovary with access efficiency while improving pollination accuracy, suggesting that variation in herkogamy traits is a trade‐off between the visitation frequency and pollination accuracy, which may be an adaptive strategy when pollinators are scarce.

The evaluation of the breeding system indicates that automatic selfing cannot occur in *E. fischeriana* and that the species is not pollen limited under natural conditions (Figure 5). This may be due to herkogamy throughout the flowering period; the female flower straightened in female phase (vertical herkogamy) but reflexed in the male phase (lateral herkogamy). The frequency of visits and pollinator capacity to deposit pollen both have a role in how important floral visitors are to plant reproductive success (Fenster et al., 2004; Thomson, 2003). Our results indicate that both herkogamy traits in *E. fischeriana* are phenotypically variable, and show both vertical and lateral types. It is also suggested that the type of herkogamy had direct effects on pollen removal and deposition and seed set, and also affects the attraction to pollinators. The seed set ratio and pollen deposition of vertical herkogamy were significantly higher than lateral herkogamy, and lateral herkogamy may promote pollen removal. In addition, it has been proposed that the consecutive expression of two types of herkogamy (lateral and vertical herkogamy) in the same flower may be strongly selected by differences in pollination environments (Herlihy & Eckert, 2007; Jiménez‐López et al., 2019; Takebayashi et al., 2006). Our results support this hypothesis by showing that pollination can significantly promote bending of gynophore, which in turn can lead to variation in the type of herkogamy (Figure 8). When the number of available pollinators across a population is low (pollen limitation), an increase in the female phase (lateral herkogamy) time can promote pollen deposition, the rate of outcrossing, and increases the diversity of different outcrossing pollen genotypes at the stigma by an extended period for pollinator visitation (Delph & Havens, 1998; Jorgensen & Arathi, 2013; Van Doorn, 1997),.According to this result, selection through female function for traits that increase pollen deposition and post‐pollination pollen success should vary positively with the intensity of pollen limitation, an expectation confirmed for traits involved in pollinator attraction (Ashman & Morgan, 2004), whereas in higher availability of pollinators, a fast conversion in types of herkogamy promoted pollen output. This suggests that the mechanism primarily helps *E. fischeriana* to overcome pollen limitation and avoid inbreeding depression, while also improving male and female fitness, respectively, through the bending of the gynophores. As a result, variation in herkogamy through gynophore bending in *E. fischeriana* may be an evolutionary key trait strongly selected by changing environmental conditions and availability of pollinators.

## AUTHOR CONTRIBUTIONS

**Xiang Zhao:** Data curation (supporting); formal analysis (equal); visualization (equal); writing – original draft (equal); writing – review and editing (lead). **Guang Yang:** Data curation (supporting); formal analysis (equal); visualization (equal); writing – original draft (equal); writing – review and editing (supporting). **Qin‐zheng Hou:** Conceptualization (equal); data curation (equal); formal analysis (equal); funding acquisition (lead); writing – original draft (supporting); writing – review and editing (equal). **Wenrui Min:** Formal analysis (supporting); visualization (supporting). **Tai‐Hong Wang:** Formal analysis (supporting). **Xiaoyan Bao:** Visualization (supporting).

## FUNDING INFORMATION

This work was supported by the National Natural Science Foundation of China (31860051, 31360044, 32260054).

## CONFLICT OF INTEREST

None declared.

## Data Availability

The raw data used in the present study are available from the Dryad Digital. Repository: https://doi.org/10.5061/dryad.dbrv15f47.
